# Minimal Thermal Requirements for Development and Activity of Stored Product and Food Industry Pests (Acari, Coleoptera, Lepidoptera, Psocoptera, Diptera and Blattodea): A Review

**DOI:** 10.3390/insects10050149

**Published:** 2019-05-23

**Authors:** Vaclav Stejskal, Tomas Vendl, Zhihong Li, Radek Aulicky

**Affiliations:** 1 Crop Research Institute, Drnovska 507/73, CZ-16106 Prague 6-Ruzyne, Czech Republic; vendl@vurv.cz (T.V.); aulicky@vurv.cz (R.A.); 2 Key Laboratory of Ministry of Agriculture for Monitoring and Green Management of Crop Pests, Department of Entomology, College of Plant Protection, China Agricultural University, Beijing 100193, China; lizh@cau.edu.cn

**Keywords:** temperature, development, individual, populations, thresholds, respiration, walking, flying, forensic entomology, pest management

## Abstract

Low temperatures play an important role in arthropods because they affect both the individual and population development of all physiological and behavioural activities. Manipulation with low temperatures is a primary nonchemical pest control method. For stored product and food industry practitioners, a knowledge of pest thermal requirements, in particular threshold temperatures at which development and other activities of a particular pest species cease, is of crucial importance. This review presents summary data regarding the lower temperature thresholds of 121 species of stored product and food industry pests from six arthropod taxa (Acari, Coleoptera, Lepidoptera, Psocoptera, Diptera, and Blattodea). In particular, this review collected and summarized information regarding the lower development thresholds, lower population thresholds, lower acoustic or respiratory thresholds, lower walking and flying thresholds and lower trap capture thresholds for flying and walking arthropods. The average lower development threshold (LDT) differed among orders: the lowest was reported for Acari (6.8 °C) and Diptera (8.1 °C), followed by Lepidoptera (11.3 °C) and Psocoptera (13.8 °C), and the highest was reported for Coleoptera (14 °C) and Blattodea (15 °C). An exclusion-function was established showing the percentage of pest species (*n* = 112) that were developmentally suppressed (excluded) due to temperatures reaching the LDT in the range of decreasing temperatures from 25 °C to 0 °C. We scaled various temperature thresholds from the lowest to highest temperature as follows: the walking threshold, the trap capture threshold for walking insects, the lower development threshold, lower population threshold, lower flying threshold and the lower trap capture threshold for flying pests. Important pest species were identified for which information regarding the lower temperature threshold is missing, or for which the information is too variable and should be refined in future research.

## 1. Introduction

Commodity stores, food processing facilities and distribution chains can be infested and contaminated by many species of pest [1,2,3], mainly acaroid mites and certain insect orders (Lepidoptera, Coleoptera, Psocoptera, Blattodea, Diptera). In recent decades, there has been an increasing demand for the use of non-chemical methods for the control of these pests, among which temperature manipulation has played a prominent role. This is not surprising because temperature is one of the key environmental factors affecting the physiological, life history, behavioural and population processes of arthropods. Techniques of manipulation with low temperatures play a more important role in commodity and finished food storage than in field and glasshouse agriculture. This is mainly because commodities are stored in enclosed facilities, technically enabling temperature manipulations, and grain seeds are stored in the quiescence phase. In contrast to field pests, some stored product beetles may increase the temperature of the grain due to their intensive respiration during the entire storage season [4,5]. The latter phenomenon increases the demand for proper temperature management in order to maintain low temperatures during commodity storage.

It is well known that with decreasing/increasing temperature, arthropod development gradually slows down, and at a certain point stops completely. The temperature range between the minimum and maximum is known as the “thermal window” [6]. The relationship between temperature and arthropod development can be mathematically described by a variety of so-called temperature performance curves [7]. Adequate specific lower and upper temperature thresholds and performance curves were estimated for various physiological processes and behavioural activities (e.g., walking, flying, respiration). Because arthropod activity performance is a function of body temperature, an increasing temperature generally supports higher physiological activity in arthropods [8]. However, the temperature performance curve of the arthropod behavioural response to temperature is not always simply shaped as is the case for the developmental rate. For example, locomotion rates of adult *Sitophilus oryzae* (L.) and *S. zeamais* (Motschulsky) increased almost linearly between 5 and 30 °C [9], whereas *Ptinus tectus* showed greater activity at fluctuating temperatures between 10 and 20 °C than at a constant 25 °C [10].

Although the above-described relationship between the developmental rate and temperature has general validity for all arthropods, there are substantial differences in the absolute values of specific minimum/maximum thresholds and the breadth of temperature windows among various strains, species and higher taxa (e.g., [11]). Interspecific thermal variability among stored product pests was described early in the last century in various *Sitophilus* species [12,13]. Beckett [14] illustrated that particular stored product species show a different range of conditions that favour their development and population growth with the following contrasting examples: “Cooler moister condition tends to favour *S. oryzae*, warmer moister conditions favour *Cryptolestes ferrugineus* (Stephens), while warmer drier conditions favour *Tribolium castaneum* Herbst”. Interspecific differences in behavioural reactions, such as locomotion and flying activity, in response to low temperature may be expressed even more profoundly than temperature development thresholds. For instance, cold-adapted species may walk at near zero or sub-zero temperatures (−16 °C) [15], while in tropical species, their lowest walking thresholds may be as high as 15 °C [16]. Some stored product insect pests were documented to walk at low temperatures (2.5 °C), while other species could not move until temperatures reached 10 °C or higher [17,18].

Due to the importance of low temperatures in control programs for the safe storage of commodities and finished products, agricultural and food safety practitioners need summarized information about temperatures relevant to pest control, monitoring and pest risk modelling. Regarding stored product pests, the majority of studies are focused on the effect of extreme temperatures on mortality and population growth [19,20,21]. There are several general papers [11,22,23] and databases [24] which review the minimal temperature requirements for arthropod development that also include selected stored and food product pests. The only specialized review on the lower development thresholds of stored and food industry pests was prepared by Imura [25] and the only review on lower temperature thresholds for population development was prepared by Howe [26]. Some data were summarized in a textbook by Hagstrum and Subramanyam [27]. However, the published resources are not updated, and some of them did not cover storage mites (Acari) and food industry pests (Blattodea, Diptera). Therefore, this review focuses on summarizing data from old reviews with the addition of newly published data on all minimal thermal requirements for individual and population development. This review does not cover stored product insect and mite responses to optimal or extreme temperatures, cold acclimation and hardiness because an overview of this topic is available (e.g., [21,28]). Instead, because of increasing interest in using traps and electro-acoustic methods for detection, there is included information regarding the availability of minimal temperature thresholds for the activity of pest arthropods, i.e., walking, flying, sound production, respiration. This review is also a first attempt to compare the relative lower development and lower activity thresholds of stored product and food industry arthropod pests.

## 2. Minimum Thermal Requirements—Terminology, Concepts and Sources of Variability

The initial sub-chapter attempts to briefly summarize the concepts and ideas used in this review and comment on sources and reasons for the variability of thermal constants.

### 2.1. Temperature Constants, Thresholds and Chill Coma Terminology and Concepts

#### 2.1.1. Lower Development Threshold (LDT), Lower Theoretical Temperature Threshold, and Lower Population Threshold (LPT)

A lower development threshold (LDT; T_0_) is defined as the temperature at which development stops [11]. Other authors use synonym terms such as “low development threshold temperature” (T_0_) [29] and “minimal temperature for development” (T_min_) or “minimum temperature needed for development to occur” (T_min_) [30]. Rebaudo and Rabhi [7] discriminate two types of LDT while describing the temperature performance curve: an LDT obtained by a nonlinear model is termed a “critical thermal minimum (CT_min_)”, and an LDT obtained by straight-line models is termed a “Tbase temperature”. Although Beckett [14] used an LDT for stored product insects with the more precise term “theoretical threshold of immature development”, in this review, it was decided to use the term “lower development threshold” or its acronym “LDT” because it is more frequently used in the current literature. The remaining terms and acronyms may be confused with some similar ideas, e.g., those used to describe physiological states related to chilling points such as CT_min_. Here, it is relevant to note that LDT is frequently associated with another temperature constant named the “sum of effective temperatures” (SET), which is defined as the number of heat units (called day degrees, dd, DD) required to complete development of a stage. Réaumur [31] was among the first scientists to observe that the development of poikilothermic organisms is limited by specific low temperatures and that the temperature sum needed to complete development tends to be constant. The inverse relationship between the SET and the LDT for development has been described by Honek and Kocourek [32]. Although the SET is used for phenological and forensic modelling due to size limitations, this thermal constant is not used in this review. The LDT is a marginal value for insect development, and such extremes may be associated with high mortality. As such, LDT values may not necessarily be able to support even a minimal sustainable population or its increase. Therefore, some authors recommend estimating the so-called “lower population threshold” (lower threshold for population development) (LPT) instead of estimating the LDT. This is the lowest temperature at which arthropods multiply and their population is able to at least minimally increase. It is why Beckett [14] used the LPT synonym “minimum theoretical threshold temperatures for population increase”. Howe [26] and Sinha [33] were the first to summarize the LPT (using the ideas of “minimal conditions for population increase” and “approximate minimum at which major storage mites breed”) for stored product insects and mites. Beckett [14] stressed that the LDT is lower than the LPT, and Howe [26] estimated that the LPT values are approximately 3–5 °C above LDT values in most storage arthropod pest species. Minimum population thresholds may be less precise in estimation—due to their inherent higher complexity—than the LDT, but the LPT is more convenient for usage in storage practice, especially for creating strategies to protect stored grains from pest population increases by cooling and ventilation [34].

#### 2.1.2. Cold Hardiness, Chill Coma, Critical Thermal Minimum and Cctivity Thresholds (Flying, Walking Respiration)

Low temperatures affect not only individual or population development but also the activity of arthropods (movement, respiration, sound production, etc.) and their survival [21]. The most general term regarding tolerance to low temperature is “cold hardiness”. Andreadis and Athanassiou [28] defined cold hardiness simply as the capacity of arthropods to survive low temperature exposure. There is extensive research on understanding at what low temperatures (upper cooling points /SCPs/, lower lethal temperatures/LLT) irreversible changes leading to death of arthropods occur and the main physiological adaptations to tolerate extremely low temperatures. Well above the instantly killing extreme temperatures, there is a range of suboptimal temperatures associated with the inhibition of arthropod activities that include movement, physiological processes and neuromuscular transmission. Such a range of states, which are reversible if exposure is not too long (i.e., [35]), are usually described as chill coma (T_cc_ or CT_min_) [36]. Coombs and Bale [37] described the critical thermal minimum (CT_min_) and chill coma as “nonlethal values of the lowest temperatures at which an invertebrate can perform motile tasks. Providing that the exposure temperature increases, invertebrates are usually able to regain the use of their limbs (chill coma recovery) and eventually to walk in a coordinated manner (activity recovery)”. There are attempts to precisely describe states approaching chill coma (e.g., [18]), to create subtle grading for the various degrees of chill coma, or to include aspects of spontaneous vs. disturbed reactions at low temperature. This is certainly useful for a particular experiment, but it leads to terminological proliferation and, without general consensus, ambiguity. One such term is the “critical thermal minimum” (CT_min_). Some authors understand the CT_min_ as the lowest temperature at which it is possible for a species to walk in a coordinated way (e.g., [37,38]), while others (e.g., [39]) consider the CT_min_ the temperature at which exposed arthropod individuals are not able to move even when stimulated or irritated. Hazell et al. [16] avoided terminological ambiguity using the term “activity threshold” for the highest temperature at which each insect individual last spontaneously walked and chill coma for a state determined by the last movement of its legs or antennae. When studying the minimum temperature at which stored-product insects can move inside stored grain bulks, Jian et al. [18] established three specific terms. The first term was “spontaneous walking stops” (SM_h_), which was defined as “the highest temperature at which insects stopped spontaneous movement”. Jian et al. [18], unlike other authors [36], do not consider SM_h_ to be an upper marginal value of the chill-coma zone with the following substantiation: “insects in these states (i.e., SM_h_) can resume moving when they are disturbed during grain loading and unloading… The SM_h_ is important for the grain industry because insects might be able to move to other locations and storage facilities during transportation if the grain and/or ambient temperatures are higher than T_cc_, CT_min_, or CCT”. The second term, “no movement after shaken” (CCT), was defined as “the highest temperature at which insects lack antenna and/or leg movement under disturbed conditions”. This is an equivalent to what other authors (e.g., [39]) termed the “critical thermal minimum” (CT_min_). The third term was the “temperature movement minimum” (TM_min_), which was estimated as the minimal temperature at which it was possible to capture the tested pest adults into a pitfall trap. However, using trap capture as criteria of minimum walking ability may not always be quite accurate because the distinct species of stored product insects differ profoundly in their behavioural response to various pitfall traps even at identical temperatures (e.g., [40,41,42]). To avoid confusing terminology regarding activity thresholds, this review followed the approach proposed by Hazell et al. [16] and Wakefield and Cogan [40] and decided to use simple intuitive terms as follows. The terms “flying thresholds” and “walking thresholds” were used for data extracted from published experiments that are based on direct observation of behaviour of undisturbed individuals [17,18,43,44]. The terms “trap capture thresholds for flying pests” and “trap capture thresholds for walking pests” were used for data extracted from published laboratory or field experiments where captures into traps were associated with some minimal temperatures or thresholds (i.e., [18,45]).

### 2.2. Inter- and Intra-Species Sources of Variability for Thermal Constants

#### 2.2.1. Taxon, Species, Strains, Individuals and Thermal Adaptation

Multiple reviews (e.g., [11,26,29,46]) have documented that thermal thresholds (LDT; LPT) differ profoundly among various higher taxa and species. It was suggested that the LDT is affected by geographical origin as a result of adaptation to specific conditions. Trudgill [47] observed that tropical poikilothermic organisms tend to have higher threshold temperatures for development than temperate organisms. Honek [11] compared LDT values according to the origin of species across multiple insect orders: the average LDT for tropical species was 13.65 °C, for subtropical species it was 10.46 °C, and for temperate species it was 7.89 °C. There is also a certain level of intrinsic natural variation of developmental rates among individuals within strains and populations [48]. There may also be differences between strains from identical species collected in different geographical regions [49,50,51]. Bergant and Trdan [52] suggested differences between strains adapted to laboratory and field conditions. However, Subramanyam and Hagstrum [53] found minimal effects of various strains of the stored product moth *Ephestia cautella* on temperature constants.

#### 2.2.2. “Rate Isomorphy” and Identical LDTs in Developmental Stages of a Species

The published reviews [23,29,54] show that LDTs are not always equal when estimated for specific stages such as egg, larva, pupa, and for the entire egg to adult period. This implies a question regarding whether it is always necessary to measure all stages to obtain proper information regarding characteristic LDTs for a particular species. Jarosik et al. [55] described a new ecological phenomenon called “rate isomorphy”. It says that with changing temperature, the developmental stages of a population of a species take constant proportions of the total development time. They found that the validity of rate isomorphy was confirmed in 57% of the studied populations of different insect species. It can be derived from rate isomorphy that development stages appear to have the same (population-specific) LDT, which is of practical importance: the LDT established for one developmental stage, preferentially one that is easy to handle, could be used for predicting the other stages.

#### 2.2.3. Thermal Acclimation

Most acclimation-related publications are focused on chill coma and survival at suboptimal or extremely low temperatures (e.g., [21,35,56,57]) rather than on LDT or activity thresholds [16,18]. Although there is not abundant literature on the subject, it is sufficient to show that thermal acclimation greatly affects activity thresholds. Hubert et al. [58] found effects of acclimation on mite respiratory activity and behavioural temperature preference. Evans [59] observed different patterns of respiration (measured as oxygen consumption) of acclimatized and non-acclimatized individuals of *S. oryzae* and *S. granarius*. However, because it was not able to find a paper documenting an effect of acclimation on the LDT in various stored product arthropod individuals originating from an identical population, that information is not included in Table 1, Table 2, Table 3, Table 4, Table 5 and Table 6.

#### 2.2.4. Environmental Factors (Humidity, Moisture, and Food Quality)

There is documentation that both the development and activity of arthropods may be, in addition to temperature, more or less affected by various environmental factors. Honek et al. [60] found that thermal requirements for completing a particular developmental stage of field Lepidoptera may vary according to the available food quality. Hagstrum and Milliken [61], in their general Coleoptera comparative study, ordered various environmental factors according to the strength of influence on development as follows: temperature > moisture > diet. Subramanyam and Hagstrum [53] found that the egg to adult development of stored product moths (*Plodia* sp., *Ephestia* spp.) was not affected by the range of lower humidity (up to 60% RH), but it was significantly affected by higher humidity (85%–95% RH) at which development was faster. In Coleopteran stored product pests, the identical conditions of food moisture and ambient humidity may have different effects in different species [62]. For example, the LDT was significantly lower when *C. ferrugineus* and *Tribolium* spp. were fed diets of < 12% moisture content compared to diets of > 12% moisture content, while this was reversed in *Oryzaephilus surinamensis*.

#### 2.2.5. Models

Temperature and development can be described by several models [7,63] that can be broadly classified into two categories: linear and non-linear. Linear models (e.g., [7,49,64]) are simple to calculate and directly show so called temperature constants (i.e., LDT and SET). Non-linear models are more numerous (e.g., [7,65,66,67,68]), and some of them also show temperature constants (i.e., LDT, SET and upper developemt thresholds) of development. Nonlinear models are used not only for modelling individual development (LDT) but also for modelling lower population thresholds (LPT) (e.g., [46]). The main distinction between linear and non-linear models is that the non-linear form may provide an optimum temperature and enable the incorporation of the effects of non-linearity at low and high temperatures. As a result, the LDT calculated using non-linear forms tends to be lower than the LDT calculated using the linear model. Because the type of model may be a significant source of variability data was included, where available, in Table 1, Table 2, Table 3, Table 4, Table 5 and Table 6 regarding which type of model was used.

#### 2.2.6. Experimental Design and Errors

Before using the data in both theoretical and practical models, the main challenge is distinguishing between natural variation of LDT values and variation caused by experimental bias [54]. Bergant and Trdan [52] asserted in their study that the particular experimental setup may be an important source of uncertainty for establishing LDT-based models. They notably mentioned that field and laboratory experiments may yield different results. However, it can be expected that for storage and food industry pests, which are mainly indoor pests, the difference will not be of such importance as in field/orchard pests. Because of this uncertainty, a range of values is usually present in the Table 1, Table 2, Table 3, Table 4, Table 5 and Table 6 accompanied by a particular reference enabling the detailed inspection of the original resources.

## 3. Importance of Low Temperatures for Stored Product Pest Detection and Control

### 3.1. Predictive Phenological or Forensic Models (Degree-Day/DD/ = Accumulated Degree Aays/ADD/)

The knowledge of species-specific data regarding the lower development threshold and sum of effective temperatures can be used for predictive modelling of population and individual development [54]. Degree-day-based models are currently more employed by field than stored products entomologists because the latter tend to employ more complex forms of predictive models (e.g., [27]). However, DD models are increasingly used in the field of legal/forensic entomology for crime investigation [69], notably in estimating the so-called post mortem interval (PMI_min_). This trend is only beneficial from the interdisciplinary perspective because many forensically important Coleoptera and Diptera are also important storage, food and feed pests (e.g., *Necrobia* spp., *Dermestes* spp., *Trogoderma* spp., *Attagneus* spp., *Calliphora* spp., *Lucilia* spp., *Sarcophaga* spp., etc.). In addition, some authors [70] consider stored product entomology as part of forensic entomology in situations where origin and time of food infestation are used to perform legal investigations due to customer complaints or arbitration procedures.

### 3.2. Safe Commodity/Finished Food Storage and Production at Low Temperature

Apart from drying, decreasing temperature is a fundamental concept for the pest-free storage of commodities without pesticides. There are multiple research papers and reviews regarding active aeration and cooling systems in silos and flat grain stores and how to quickly, evenly and economically decrease temperature in commodity masses (e.g., [14,34,71,72,73]). Aspaly et al. [46] proposed a model based on LPTs that shows a safe storage period during winter and spring seasons. Food industry safety concepts include, e.g., within an extended framework of HACCP, systems that ensure that finished food is not stored under risky temperature conditions allowing invasion and development of a population of hazardous agents. For example, Trematerra and Fleurat-Lessard [74] suggested cooling the food production area by air conditioning below the lower threshold of motion of flying insects (i.e., *Plodia interpunctella* and *Stegobium paniceum*). They claim that below this lower limit (i.e., 14–15 °C), insects remain quiet and do not lay eggs on the produce, such as biscuits, before their wrapping and packaging.

### 3.3. Control (Long-Term Exposure)

Survival of extremely low temperatures by stored product beetle pests is associated with their cold acclimation, the relative humidity of the air and the commodity moisture content. Extremely low temperatures (i.e., frost) are known to kill sensitive and non-acclimatized pests in hours or days (e.g., [21]). Extreme temperatures are rapidly effective but can be economically and technologically demanding. However, it was recognized that prolonged exposure to suboptimal temperatures may provide substantial levels of control within weeks or months [14,75]. For example, Evans [35,57] and Beckett [14] documented that maintaining low temperature (9–13.5 °C) and humidity for 3–6 months caused 99% mortality in several species of stored product Coleopteran pests (e.g., *C. ferrugineus*, *O. surinamensis*, *Rhyzopertha dominica*, *S. granarius*, *S. oryzae* and *T. castaneum*). Experiments by Renault et al. [76] revealed that for *Alphitobius diaperinus,* the temperature of 6 °C was progressively lethal (100% chill-coma after 12 days and mortality after 22 days), while at 10 °C, 70% of the insects were alive after one month.

### 3.4. Pest Risk and Invasion Modelling

The potential geographical distribution of pests and invasive arthropods is modelled by different approaches, some of which include temperature as a key factor (e.g., [77]). Recently, it has been proposed that lower development thresholds may be used for pest risk assessment and characterization of the invasive ability of pests [78]. Invasive species have higher LDTs and lower sum of effective temperatures than those never recorded outside their native ranges.

### 3.5. Efficacy of Fumigants and Modified Atmospheres and Low Temperature

Bond [79] stated that most insecticidal fumigants are used at temperatures ranging from 10 to 35 °C and that an increase in temperature positively affects fumigant efficacy. For example, phosphine may not provide 100% mortality of *T. confusum* eggs during mill fumigation even at moderately low temperatures (19.6–20.4 °C) [80]. According to Navarro [81] to achieve sufficient insect control under the hypoxic conditions of controlled atmospheres (i.e., pest exposures at low O_2_ and high CO_2_/N_2_ concentrations and in a reasonable time period), the temperature of the treated grain commodity should be elevated above 21 °C. Boardman et al. [82] summarized the physiological mechanisms involved in low temperature tolerance and controlled atmosphere interactions. They reported complex mechanisms that either improve or reduce insect survival under a combination of controlled atmosphere and low temperature treatments. Some fumigants may passively and slowly penetrate into pest bodies, but it appears that the main and rapid entrance route of fumigants and inert gasses is the respiratory system [79]. Although it is known that respiration intensity generally correlates with temperature [59,83], there is little published information regarding pest respiratory activity at low temperatures [58].

### 3.6. Activity-Based Detection and Monitoring (Traps/Acoustic Methods)

The decision-making process, within a framework of IPM (Integrated Pest Management), is based on early detection and regular monitoring of pests in commodity stores and food industry production facilities and distribution chains. A variety of traps are available for monitoring. However, the usage of all types of traps is limited by the presence of sufficiently high temperatures because they rely on the flying or walking activity of insects and thus on ambient temperature. Field experiments [84] revealed that pitfall trap capture decreased as grain temperature decreased, even though pest density remained unchanged. Jian et al. [18] showed that there are species-specific low threshold temperatures at which pests are not caught in traps. Recently, acoustic methods have been proposed for use in discovering internally feeding pests (e.g., [85,86,87]), which is also strongly dependent on the feeding activity of insects. Mankin et al. [87] documented that thermal treatment can significantly increase the sensitivity of acoustic detection of hidden infestations of stored grain by larvae of *S. oryzae*.

## 4. Lower Development and Population Thresholds in Various Taxa

In total, data on LDTs and LPTs for 121 selected (i.e., by the highest importance) stored product arthropod species from Acari and 5 insect orders were obtained. The data are summarized in Table 1, Table 2, Table 3, Table 4, Table 5 and Table 6. Most of the data were gathered from several general papers, reviews and databases dealing with temperature constants/thresholds (e.g., [22,24,25,26]). Data for the species that were not included in these studies were searched separately in original articles and included in the summary tables and lists. Important species on which it was not able to find either LDT or any of the focal variables are mentioned in the corresponding chapters. Based on our survey, it seems that there are also missing data on some important food industry pest species from other insect orders, such as *Lepisma saccharina*, *Thermobia domestica* (Zygentoma), *Acheta domesticus* (Orthoptera) and *Monomorium pharaonis* (Hymenoptera).

### 4.1. Minimal Thermal Requirements for Development of Mites (Acari)

**Figure 1:**
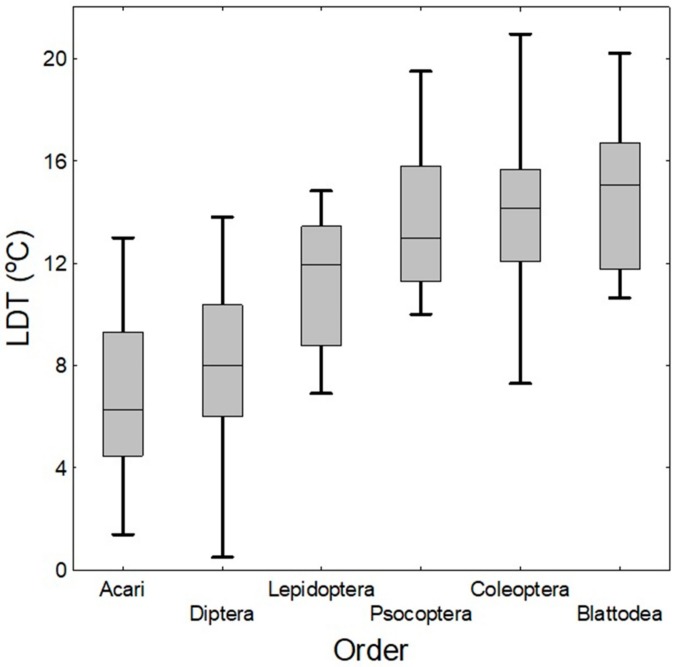
Boxplots of LDTs in all orders. Median of LDT for Acari = 6.28 °C, for Diptera = 8.0 °C, for Lepidoptera = 11.96 °C, for Psocoptera = 13.0 °C, for Coleoptera = 14.15 °C, and for Blattodea = 15.05 °C. (Horizontal lines inside the boxes represent median values, upper and lower boxes represent the 75th and 25th percentiles, respectively, and upper and lower whiskers represent the 99th and 1st percentiles, respectively).

The overview of lower development (LDT) and population (LPT) thresholds is presented in Table 1. Pooled data from both thresholds were obtained for 15 species from 10 genera (e.g., [33,46]). In mites, the average value for the LDT was 6.8 ± 1.1 °C (Figure 1), and for the LPT, it was 11.4 ± 1.2 °C. The minimal and maximal LDTs were 1.4 °C and 12 °C, respectively, while the minimal and maximal LPTs were 5 °C and 22 °C, respectively.

### 4.2. Minimal Thermal Requirements for Development of Beetles (Coleoptera)

Lower development (LDT) and population (LPT) thresholds are summarized in Table 2. Pooled data from both thresholds were obtained for 55 species from 30 genera (e.g., [14,22,24,25,26]). Nevertheless, for some of these species (namely, *Gnatocerus cornutus*, *Necrobia rufipes*, *Niptus hololeucus*, *Ptinus fur* and *Tipnus unicolor)*, it was able to find only LPTs, but not LDTs. Moreover, for some important species (e.g., *Attagenus* spp., *Bruchus pisorum*, *Caryedon gonagra*, *Cathartus quadricollis*, *Mycetophagus* spp., *Reesa vespulae*, *Tenebrio* spp. and *Tenebroides mauritanicus*), it was not able to find either of the two focal variables. The average value for the LDT was 14.0 ± 0.4 °C (Figure 1) and for the LPT it was 18.9 ± 0.6 °C. The minimal and maximal LDTs were 7.1 °C and 22.8 °C, respectively, while the minimal and maximal LPTs were 10 °C and 26 °C, respectively. The data revealed high variability between individual species as well as between families: for example, Ptinidae is the family with the lowest mean LDT (11.9 °C), whereas Tenebrionidae is on the opposite end of the scale (16.5 °C). In some cases, where more values for a single species are available, a high variability on the intraspecific level was observed. This particularly concerns *Carpophilus hemipterus*, *Oryzaephilus surinamensis* and *Trogoderma granarium*. Such variability can be caused by various factors discussed in Section 2.2.

### 4.3. Minimal Thermal Requirements for Development of Moths (Lepidoptera)

An overview of the lower development (LDT) and population (LPT) thresholds of Lepidoptera is presented in Table 3. Pooled data from both thresholds were obtained for 16 species from 13 genera (e.g., [22,24,25,53]). For one species (*Sitotroga cerealella*), data only on the LPT, but no on the LDT, were found. The average LDT value was 11.3 ± 0.7 °C (Figure 1), and the average LPT value was 14.9 ± 1.2 °C. The minimal and maximal LDTs were 6.9 °C and 21.4 °C, respectively, while the minimal and maximal LPTs were 10 °C and 18 °C, respectively. Similarly as in Coleoptera, there is high intraspecific variability in some pests, which is reflected by the inclusion of more records for identical species in Table 3. This concerns *P. interpunctella* and mainly *Cadra calidella*, where the range of the LDT is enormous and should thus be treated with caution.

### 4.4. Minimal Thermal Requirements for Development of Psocids (Psocoptera)

An overview of the lower development (LDT) and population (LPT) thresholds are summarized in Table 4. Data for lower development thresholds were obtained for 7 species of the genus *Liposcelis* (e.g., [24,111]). The average value for LDT was 13.8 ± 1.2 °C (Figure 1), and for LPT, it was 19.5 ± 1.4 °C. The minimal and maximal LDTs were 8.17 °C and 20.9 °C, respectively, while the minimal and maximal LPTs were 15 °C and 21 °C, respectively. Nevertheless, LPT data were obtained for only two species.

### 4.5. Minimal Thermal Requirements for Development of Cockroaches (Blattodea) and Flies (Diptera)

Lower development thresholds (LDT) are summarized in Table 5 and Table 6. The data were obtained for 7 Blattodea species from 3 genera and for 21 Diptera species from 15 genera (e.g., [22,24]). The average LDT value was 15 ± 1.2 °C (Figure 1) for Blattodea and 8.1 ± 0.6 °C for Diptera. For Blattodea, the minimal and maximal LDTs were 7.02 °C and 20.2 °C, respectively, but for Diptera these were 0.5 °C and 15.39 °C, respectively. For both orders, no data were found on LPTs. In the case of Blattodea, there are some important food industry pest species on which no data were found on LDTs (especially *Blatta orientalis* and *Supella longipalpa*).

### 4.6. Comparison of LDTs of Taxa and Temperature Exclusion Function

**Figure 2:**
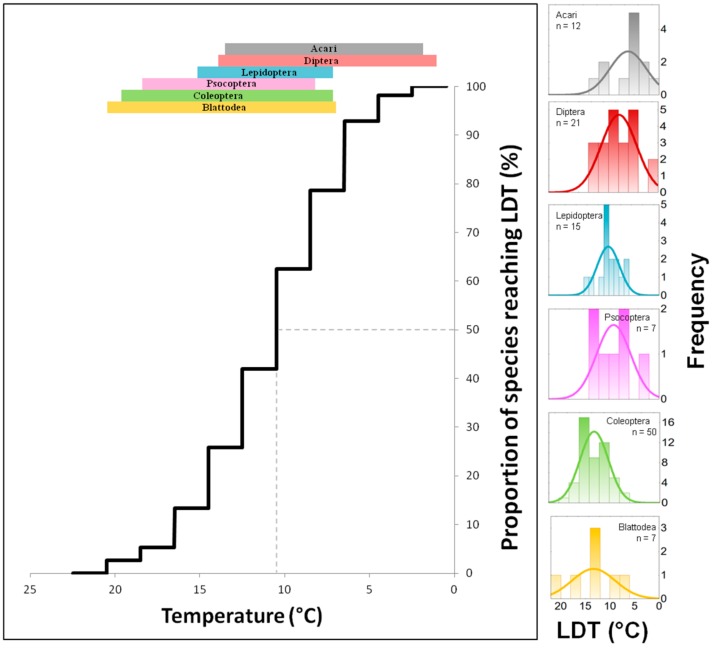
Exclusion function: Percentage proportion (*n* = 112) of pest species (from Acari and 5 insect orders) that were excluded from development due to reaching a lower development threshold (LDT) at a particular decreasing temperature. Colour bands on the top of the figure show the particular temperature range of LDTs for each order. On the right-hand side of the figure, there are frequency histograms of LDTs for each order.

The comparison of data from Table 1, Table 2, Table 3, Table 4, Table 5 and Table 6 shows profound differences in LDT values not only among various species as expected but also among the average values obtained for entire orders. The lowest average LDTs were found for Acari and Diptera, whereas Psocoptera, Coleoptera and Blattodea have LDTs approximately 6 °C higher (Figure 1). Data show that the efficacy and effectiveness of ventilation/cooling differ for different ranges of temperatures. For example, Figure 2 illustrates the effect of decreasing temperature on the proportion of stored product and food industry pest species (pooled data from Table 1, Table 2, Table 3, Table 4, Table 5 and Table 6) that are excluded from development due to reaching the LDT at particular temperatures. It is apparent that decreasing temperature by (arbitrarily selected) steps of 5 °C has strongly nonlinear effects on the amount of excluded species. For example, the decrease of temperature from 25 °C to 20 °C excluded 4% of the pest species from development; the decrease from 20 °C to 15 °C excluded approximately 15% of the pest species, and the decrease from 15 °C to 10 °C excluded the development of more than 50% of pest species.

## 5. Lower Activity Thresholds (Flight, Locomotion, Sound Production, Respiration) and Their Relation to LDTs

### 5.1. Lower Acoustic Detection Thresholds (LAT)

There are two types of arthropod pest activities producing some sort of sound according to which pests can be detected in the infested commodity by acoustic sensors: either feeding and gnawing inside/on the grain kernels [85] or walking through the infested commodity mass [138]. Most of the information regarding the effect of temperature on sound detection of both previously mentioned types is related to high temperatures. Hagstrum and Flinn [86] observed that sounds of *S. oryzae* and *R. dominica* increased as the temperature increased from 17.5 to 30 °C, whereas sound production of *T. castaneum* was the lowest at 25 °C and then increased with increasing temperature. Fleurat-Lessard et al. [85] estimated an LDT for acoustic detection of *S. oryzae* larva at 8 °C. They noticed that acoustic detection was “lower than was previously estimated and far below (i.e., 1.63×) the theoretical thermal low limit (i.e., LDT) for *S. oryzae* development (13 °C)”. However, the potential for acoustic detection of pest feeding activity may be even lower than that. Granovsky and Mills [139] demonstrated that acclimation allows *S. granarius* feeding on damaged wheat kernels at temperatures as low as 4.4 °C. The lower acoustic threshold (8 °C) estimated by Fleurat-Lessard et al. [85] for feeding by larvae of *S. oryzae* is lower than the walking threshold for *S. oryzae* adults estimated by Jian et al. [18] (9 °C), but not lower than the LWT estimated by Wakefield [17] (5 °C). Walking sounds may also be produced by some species at very low temperatures. For example, *C. ferrugineus* can spontaneously move in grain masses at temperatures of 4–8 °C [18]. Nevertheless, the intensity of any activity at the marginal values is very low, and it is therefore challenging for the sensitivity of acoustic detectors to discriminate the weak pest-related sounds from the relatively more intense background noise.

### 5.2. Lower Respiratory Thresholds (LRT)

For stored product insects, information is available regarding respiration at optimal conditions and respiration intensity in various modified or controlled atmospheres (e.g., [140,141]. However, there are few works regarding respiration at low temperatures. For example, Hanec [142] found that the rate of respiration of *C. ferrugineus* increased as the temperature increased from 1–30 °C. Edwards [143] reported that respiration of *T. confusum*, measured as oxygen consumption, occurred even at a temperature of 5 °C, and with increasing temperature up to 34 °C, it increased 16× (i.e., 5 °C—0.16; 10 °C—0.32; 18 °C—0.81; 26 °C—1.54; 34 °C—2.63—measured as oxygen consumption mm^3^/mg/hr). He commented that respiration can be maintained (at a reduced level) even at sub-zero temperatures for a short time. Although the abovementioned works give some estimation that respiration may occur at low temperatures, it was not able to find any published data on explicitly measured lower respiratory thresholds (LRT) for stored product taxonomic groups other than mites. Hubert et al. [58] estimated the LRT in one dust mite (*Dermatophagoides farinae* Hughes-LRT—5.2 °C) and 3 stored product species, in which LRTs ranged from 0.8 to 2.3 °C (Table 7). In *Acarus siro* (L.) and *Lepidoglyphus destructor* (Schrank), the LRT values were slightly lower than their LDTs, while in *Tyrophagus putrescentiae* (Schrank) the LRT was 6.25× lower than its LDT.

### 5.3. Lower Flying Thresholds (LFT) and Lower Trap Capture Thresholds for Flying Pests (LTCT-FP)

**Figure 3:**
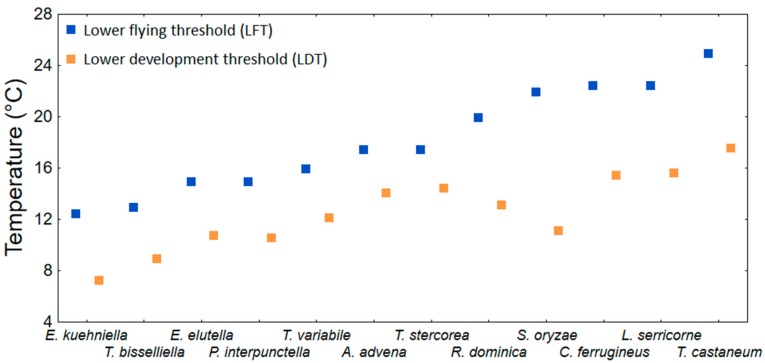
Comparison of lower flying (LFT) and development thresholds (LDT) of moth and beetle species for which both variables are available. (The lowest LDT value from Table 2 and Table 3 was used for each species).

Lower flying thresholds were estimated under laboratory conditions for several species of stored product moths and beetles [43,44,144,145]. They are summarized in Table 8. The LFT ranged from 12.0 to 27.5 °C with an average of 18.3 (± 1.3 SE) °C. Figure 3 shows, using the data from Table 8, that the LFT tends to be higher than the LDT. On average, the LFT is approximately 6.0 (± 1.0 SE) °C higher than the lowest recorded LDT for the particular species. The only inconsistent case is *S. oryzae*, in which the LFT is approximately 11 °C higher than the LDT (Figure 3). Several field studies deal with temperatures and captures of flying insects. Giles [146] analysed the capture from fly traps of *S. zeamais* and another 12 stored product Coleopteran species in Kenya. He found that sunny days affected capture in addition to temperature. In Australia, flight of *T. castaneum*, *R. dominica*, *S. oryzae*, and *Cryptolestes* spp. in the field was detected when the temperature was greater than 26 °C [147]. Several field studies have suggested that the minimum temperature at which *Sitophilus* spp. flight occurs is between 20 °C and 23.3 °C [146,148,149], while Throne and Cline [150] did not observe any *S. oryzae* and *S. zemais* in flight traps when the maximum temperatures recorded during a week were less than 23.3 °C to 26.1 °C. In Arkansas (USA) McKay et al. [151] found little flight outdoor activity of *R. dominica*, *T. variabile*, *L. serricorne* and *P. interpunctella* bellow average temeperature of 17 °C and suggested this teperature as the LTCT-FP for the observed species. The most comprehensive study, in terms of the concurrent number of pest species, was executed by Throne and Cline [45] in the USA in South Carolina. They analysed seasonal flight patterns of stored product Coleoptera species using sticky traps. Most of the captured species and individuals were found when the weekly average temperature was between 20 and 30 °C. The average lowest temperature at which the particular species (16 species) were captured was 24.7 (± 1.4 SE) °C. For the purpose of this review, the average value (24.7 °C) is the approximate value of the LTCT-FP, which was approximately 6.4 °C higher than the average LFT (Table 8).

### 5.4. Lower Walking Threshold (LWT) and Lower Trap Capture Threshold for Walking Pests (LTCT-WP)

**Figure 4:**
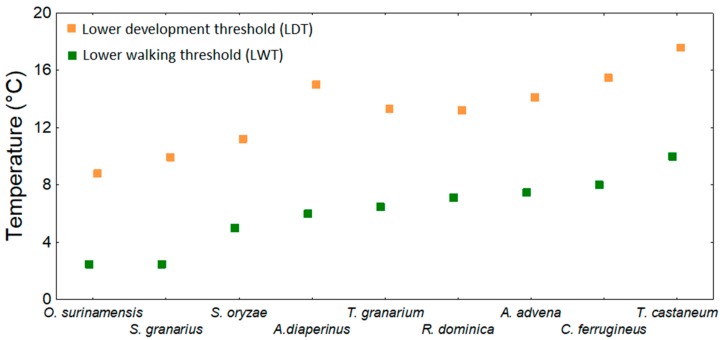
Comparison of lower walking (LWT) and development thresholds (LDT) of stored-product beetles for which both variables are available. (The lowest LDT value from Table 2 and Table 3 was used for each species).

Mellanby [8] was one of the first authors to show the LWT for adults of some food industry pests and that the LWT can be changed by previous acclimation at different temperatures (i.e., in 30 °C/14–17 °C): *B. orientalis* LWT—7.7/2.5 °C; *Lucilia serricata* LWT—6/3.5 °C; *Calliphora ertyhrocepahala* LWT—4/1 °C. Ernst and Mutchmor [154] described patterns of dispersal of three species of grain beetles (*Tenebrio molitor*, *T. confusum*, and *Trogoderma parabile*) as a function of thermal acclimation and storage temperature, and Barlow and Kerr [155] studied how temperature changes can affect the locomotion of *S. granarius*. Hanec et al. [142] observed that adults of *C. ferrugineus* cannot move at or below 2 °C. LWTs were estimated under laboratory conditions for several species of stored product beetles [17,18,76], and the lower trapping thresholds (LTCT-WP) were estimated by Jian et al. [18]. LWTs and LTCT-WPs are summarized in Table 8. Figure 4 shows, using the data from Table 8, that the LWT tends to be lower than the LDT. More specifically, the LWT is on average approximately 7.1 (± 0.3 SE) °C lower than the lowest recorded LDT.

### 5.5. Comparison of Relative Positions of Activity and Development Thresholds on a Temperature Scale

**Figure 5:**
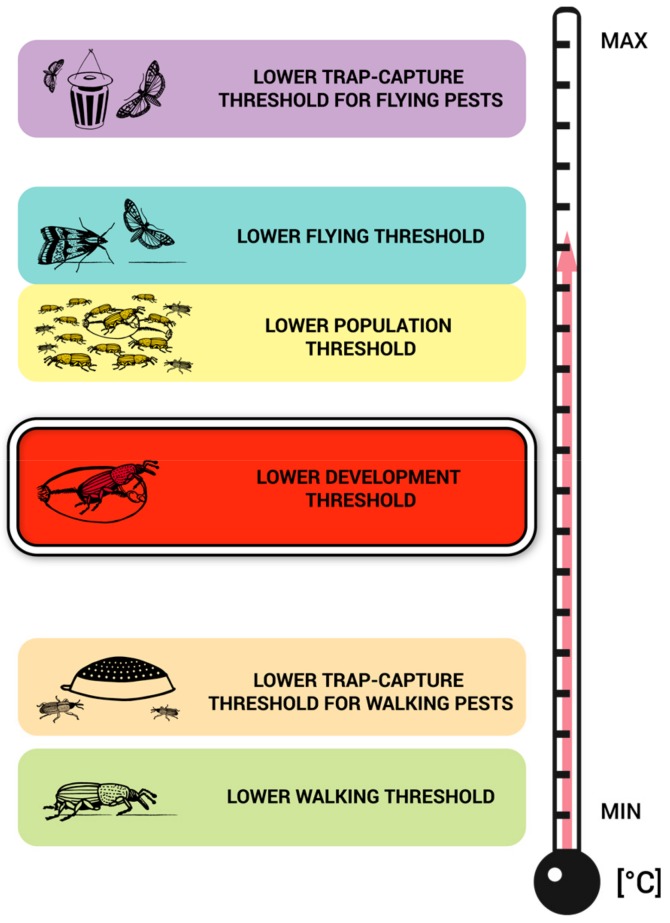
Schematic visualization of common relative positions of development and activity thresholds of stored product pests on an increasing temperature scale. Each species has different values and distances in the relative positions of these thresholds along the temperature scale. (Example of the concrete values currently available for *Cryptolestes ferrugineus*: LTCT-FP—23.3 °C, LFT—22.5 °C, LPT—22.0–23.0 °C, LDT 15.5–15.7 °C, LTCT-WP—4.0–8.0 °C, and LWT—5.0 °C).

This review shows that the most abundant published scientific data are on the lower development thresholds (LDT) and lower population thresholds (LPT), followed by data on lower walking (LWT) and lower flying (LFT) thresholds. The Figure 5 illustrated the most common relative positions of the development and activity thresholds by ordering them on an increasing temperature scale. Unfortunately, it is difficult to create such a scheme using absolute numbers for all species because the values differ greatly among species and will therefore be overlapping. However, such a scale can be constructed for each particular species separately if all threshold-related data are available. It can be exemplified using the data and case of *C. ferrugineus* as follows: LTCT-FP—23.3 °C, LFT—22.5 °C, LPT—22.0–23.0 °C, LDT—15.5–15.7 °C, LTCT-WP—4.0–8.0 °C, and LWT—5.0 °C. It is believed that this schematic figure may be useful for practitioners in two ways. First, the picture illustrates that traps for walking pests may indicate the presence of pests at such a low temperature that individuals do not develop and/or the population does not increase. In contrast, in some temperature ranges, pest population development may not be indicated by trap captures for flying pests because the flying trap-capture threshold might be above their LDTs or LPTs. Second, LDTs may serve to approximate the estimation of the remaining thresholds in cases in which the other thresholds are not concurrently known. This is due to the highest availability of LDT values in the literature and the LDT central position among the remaining thresholds at the temperature scale. However, Figure 5 should be used cautiously since there are interspecific differences; and, more importantly, acclimation and non-acclimation conditions may have a profound effect not only on the absolute values of (especially activity) thresholds but also on their relative positions. Because of the low amount of data found for particular species, lower acoustic thresholds and respiratory thresholds were not included in the schematic figure (Figure 5).

## 6. Conclusions and Suggestions for Future Research

Research on the effects of temperature on arthropods has been documented since the 18th century [31]. The accumulated scientific results have provided not only substantial insights into the physiology, ecology and geographical spread and distribution of arthropods but have also created a background for many practical applications, such as pest monitoring and control in agricultural, commodity storage, food, and industrial areas. This article provides an overview of the lower temperature development and activity thresholds for major storage and food industry pests. The study identified two main problems for future research. The first problem was missing values. For example, it was not possible to find any LDT values for the following important food industry pests: *Lepisma sacharina*, *Thermobia domestica* (Zygentoma), *Blatta orientalis* (Blattodea), and *Acheta domesticus* (Orthoptera), and stored product pests, *Attagenus* spp., *Bruchus pisorum* and *Tenebrio molitor*. Important data for particular species were also lacking for lower acoustic thresholds (most stored product arthropods) and lower respiratory thresholds (most stored product insects). The second identified problem was the high variability of already existing data for identical species, which introduces uncertainty. For example, extremely variable data are available for Indian meal moths, *P. interpunctella*, showing variations of the LDT from 10.6–18.0 °C. In the future, it would be desirable to conduct experiments leading to the completion of missing data and to conduct validation studies for already existing but highly variable data under various conditions and using different models.

## Figures and Tables

**Table 1:** Threshold temperatures for the individual (LDT) and population (LPT) development of selected stored product mites. L—estimated based on linear model, NL—estimated based on non-linear model, n.a.—data are not available.

Species	LDT (°C)	Model, r.h. (%)	Reference	LPT (°C)	Reference
*Acarus farris*	1.4	NL, 90	[88]		
*A. siro*	3.75	n.a., 70–90	[46]	5.0	[46]
	2.5	n.a.	[89]	10.2	[46]
	5.0	n.a.	[90]	7.0	[26]
*Aleuroglyphus ovatus*				10.2	[46]
				13.3	[91]
				22.0	[33]
*Carpoglyphus lactis*	5.0–10.0	n.a., 100	[92]	15.0	[33]
	4.9–7.55	L, 65–80	[93]		
*Cheyletus eruditus*				12.0	[33]
*C. malaccensis*	11.6–12.0	NL, 80	[94]		
*Dermanyssus gallinae*	4.8–5.7	NL, 65–75	[95]		
*Dermatophagoides pteronyssinus*	13.0	L, n.a.	[96]		
*Glycyphagus destructor*				10.0–15.0	[33]
*Lepidoglyphus destructor*	3.0	n.a.	[89]		
*Rhizoglyphus echinopus*	5.7	L, n.a.	[22]	6.0–10.0	[33]
*R. robini*	10.01	n.a.	[97]		
*Tyrophagus neiswanderi*	5.2	NL, 90	[88]		
*T. putrescentiae*	5.0	L, n.a.	[24]	10.4	[46]
	11.2	n.a., 98–100	[46]	10.2	[46]
	7.0–10.0	n.a.	[89]	9.0–10.0	[33]
	8.74	L, 70	[98]		
	9.1–10.4	L, n.a.	[29]		
*T. similis*	7.0	L, n.a.	[29]		

**Table 2:** Threshold temperatures for the individual (LDT) and population (LPT) development of selected stored product beetles. L—estimated based on linear model, NL—estimated based on non-linear model, n.a. —data are not available.

Species	LDT (°C)	Model, r.h. (%)	Reference	LPT (°C)	Reference
*Acanthoscelides obtectus*	11.7	L, 90	[25]	17.0	[26]
	11.1	L, n.a.	[22]		
*Ahasverus advena*	14.1	L, n.a.	[22]		
*Alphitobius diaperinus*	17.3	L, n.a.	[22]		
	15.0–17.0	n.a.	[76]		
*Anthrenus verbasci*	9.6–11.3	L, n.a.	[22]		
*Araecerus fasciculatus*	10.55	L, n.a.	[99]		
*Callosobruchus analis*	16.6	L, 70	[25]	22.0	[26]
	16.0–16.4	L, n.a.	[22]		
*C. chinensis*	10.1–10.6	L, ca. 75	[25]	19.0	[26]
	11.3–13.5	L, n.a.	[22]		
	13.9	L, n.a.	[24]		
*C. maculatus*	14.9	L, 70	[25]	22.0	[26]
	14.1–18.0	L, n.a.	[22]		
	12.98–15.0	L, n.a.	[24]		
*C. rhodesianus*	13.7	L, 70	[25]		
	11.2–15.5	L, n.a.	[22]		
	13.2	L, n.a.	[24]		
*Carpophilus hemipterus*	8.6	L, 75	[22,25]	19.0	[26]
	7.2–14.61	L, n.a.	[24]		
	14.6	L, >60	[100]		
*Cryptolestes capensis*	11.1	L, 90	[22,25]	18.0	[26]
*C. ferrugineus*	15.7	L, 70	[22,25]	20.0	[14]
	15.5	NL, n.a.	[101]	23.0	[26]
*C. pussilloides*	11.8	L, 90	[25]	18.0	[26]
	10.6	L, n.a.	[22]		
	11.0–13.0	L, 70–90	[14]		
*C. pusillus*	14.6–14.7	L, 70–90	[22,25]	14.0	[14]
	14.6–14.9	L, n.a.	[24]	22.0	[26]
	14.2	NL, n.a.	[101]		
*C. turcicus*	14.7	L, 90	[25]	21.0	[26]
	10.2	L, n.a.	[22]		
	15.0	L, 90	[14]		
*C. ugandae*	14.2	L, 90	[22,25]	20.0	[26]
*Dermestes frischii*	15.2	L, 70	[22,25]	22.0	[26]
*D. haemorrhoidalis*	13.2	L, n.a.	[24]		
*D. lardarius*	15.0	n.a.	[102]		
*D. maculatus*	15.0	L, n.a.	[101]	20.0	[26]
*Dienerella argus*	9.1–10.7	L, 75	[103]		
*Gibbium psylloides*	11.8	L, 70	[22,25]	20.0	[26]
*Gnatocerus cornutus*				16.0	[26]
*G. maxillosus*	13.3–16.1	L, n.a.	[24]		
*Lasioderma serricorne*	16.2	L, 70	[25]	18.0	[14]
	15.7	L, 70	[22]	22.0	[26]
*Latheticus oryzae*	19.8–20.6	L, 75	[22,25]	26.0	[26]
	20.6–22.8	L, n.a.	[24]		
*Lophocateres pusillus*	>15.0, <17.5	n.a., 70	[104]		
*Mezium affine*	11.2	L, 70	[22,25]	22.0	[26]
*Necrobia rufipes*				22.0	[26]
*Niptus hololeucus*				10.0	[26]
*Oryzaephilus mercator*	16.6	L, 70	[25]	20.0	[26]
	16.7	L, n.a.	[22]		
*O. surinamensis*	15.3	L, n.a.	[22]	19.0	[14]
	8.8	NL, n.a.	[101]	21.0	[26]
*Palorus ratzeburgii*	16.1	L, 70	[22,25]		
	15.4	L, n.a.	[24]		
*P. subdepressus*	15.6	L, 70	[24,25]		
	12.1	L, n.a.	[22]		
*Prostephanus truncatus*	15.1–16.2	L, 70	[22,25]		
	15.16–16.2	L, 70-90	[24]		
*Ptinus tectus*	7.1–7.5	L, 70	[22,25]	10.0	[26]
*P. fur*				10.0	[26]
*Rhyzopertha dominica*	17.5	L, 70	[22,25]	18.0	[14]
	15.1	L, n.a.	[24]	23.0	[26]
	13.2	NL, n.a.	[101]		
*Sitophilus granarius*	10.5	L, 70	[22,25]	15.0	[26]
	9.97	L, 70	[105]		
*S. oryzae*	13.5	L, 76	[25]	15.0	[14]
	12.2–13.9	L, n.a.	[22]	17.0	[26]
	11.2–15.0	L, n.a.	[24]		
*S. zeamais*	14.4	L, 76	[25]	14.0	[14]
	11.3–15.1	L, n.a.	[22]		
	13.2–13.3	L, n.a.	[24]		
*Stegobium paniceum*	13.2–14.5	L, 70	[25]	17.0	[26]
	12.1–13.9	L, n.a.	[22]		
	14.0	L, 70	[14]		
*Tipnus unicolor*				12.0	[26]
*Tribolium castaneum*	17.8	L, 70	[25]	20.0	[14]
	17.8–19.0	L, n.a.	[22,24]	22.0	[26]
	17.6	NL, n.a.	[101]		
*T. confusum*	16.7	L, 70	[25]	21.0	[26]
	15.7–16.7	L, n.a.	[22]		
	15.0–18.8	L, n.a.	[24]		
	18.3	NL, n.a.	[101]		
*T. destructor*	12.6	L, ca. 75	[25]		
	15.0	L, n.a.	[22]		
*T. madens*	17.4	L, 70	[22,25]		
*Trogoderma anthrenoides*	15.5–18.4	L, n.a.	[22]		
	18.4–18.5	L, n.a.	[24]		
*T. glabrum*	10.2–10.8	L, n.a.	[22]		
	8.2–10.8	L, n.a.	[24]		
*T. granarium*	13.3–13.9	L, 73	[22,25]	20.0	[106]
	13.5	L, n.a.	[14]	24.0	[26]
	19.0–20.8	L, n.a.	[24]		
*T. inclusum*	15.4	L, n.a.	[22]		
	11.4–12.4	L, n.a.	[24]		
*T. variabile*	12.2–15.5	L, n.a.	[22,24]		
	21.1	n.a., 30–70	[107]		
*T. versicolor*	17.1	L, 73	[22,24,25]		
	17.0	L, n.a.	[14]		
*Typhaea stercorea*	14.5	L, 80	[22,25]		
	14.9	L, 80	[24]		
*Zabrotes subfasciatus*	15.0	L, 70	[24,25]	22.0	[26]
	14.3–15.0	L, n.a.	[22]		

**Table 3:** Threshold temperatures for the individual (LDT) and population (LPT) development of selected stored product moths. L–estimated based on linear model, NL–estimated based on non-linear model, n.a.–data are not available.

Species	LDT (°C)	Model, r.h. (%)	Reference	LPT (°C)	Reference
*Apomyelois ceratoniae*	10.6	L, 70	[22,25]		
*Cadra calidella*	15.3	L, 70	[22,25]	14.0	[26]
	11.95	NL, 70	[53]		
	19.2–21.4	L, n.a.	[24]		
	9.8–10.5	L, n.a.	[24] *		
*C. cautella*	12.6	L, 70	[22,25]	17.0	[26]
	11.52	NL, 60–90	[53]		
	12.2–14.07	L, 60–85	[24]		
*C. figuliella*	12.4	L, 70	[22,25]		
	12.33	NL, 70	[53]		
	10.4	L, 70	[24]		
*Corcyra cephalonica*	14.9	L, 70	[22,25]	18.0	[26]
	14.75	NL, 70	[53]		
	13.5	L, 70	[24]		
*Endrosis sarcitrella*	8.5	L, 90	[22,25]		
*Ephestia elutella*	10.8	L, 70	[22,25]	10.0	[106]
	11.3–13.1	L, n.a.	[24]	10.0	[26]
*E. kuehniella*	7.5	L, 70	[22,25]	10.0	[26]
	8	n.a.	[102]	12.0	[106]
	9.54	NL, 60–90	[53]		
	7.3–10.9	L, n.a.	[24]		
*Hofmannophila pseudospretella*	6.9	L, 90	[22,25]		
*Nemapogon granella*	7.0	n.a., 65–95	[108]		
*Phthorimaea operculella*	11.0–13.5	L, >60	[109]		
*Plodia interpunctella*	12.1	L, 70	[25]		
	15.35	NL, 60–90	[53]	18.0	[26]
	10.6–18	L, n.a.	[24]		
	15.6–17.0	L, 60	[70]		
*Pyralis farinalis*	14.82	L, n.a.	[110]		
*Sitotroga cerealella*				16.0	[26]
*Tinea translucens*	10.0–15.0	L, n.a.	[29]		
*Tineola bisselliella*	9.0	n.a.	[102]		

* Referred to here as *Ephestia calidella.*

**Table 4:** Threshold temperatures for the individual (LDT) and population (LPT) development of selected stored product psocids. L—estimated based on linear model, NL—estimated based on non-linear model, n.a.—data are not available.

Species	LDT (°C)	Model, r.h. (%)	Reference	LPT (°C)	Reference
*Liposcelis badia*	10.0	NL, 75–80	[112]		
*L. bostrychophila*	8.17–9.16	L, n.a.	[24]		
	15.5	NL, 75–80	[113]		
	13.45	n.a.	[114]		
*L. decolor*	13.0	NL, 75–80	[115]		
*L. entomophila*	15.24–16.6	L, n.a.	[24]	18.0–21.0	[111]
	15.7	NL, 75	[116]		
*L. tricolor*	11.3	NL, 75–80	[117]		
*L. paeta*	18.1–20.9	NL, 70–80	[118]	15.0–18.0	[111]
*L. yunnaniensis*	14.77	NL, 75–80	[119]		

**Table 5:** Threshold temperatures for the individual development (LDT) of selected stored product cockroaches. L—estimated based on linear model, NL—estimated based on non-linear model, n.a.—data are not available.

Species	LDT (°C)	Model, r.h. (%)	Reference
*Blaptica dubia*	7.02–16.48	L, >50	[120]
*Blattella germanica*	15.9	L, n.a.	[22]
	16.2	L, 75	[121]
	13.7–18.5	NL, 74–76	[122]
*Periplaneta americana*	13.6	L, n.a.	[22]
	15.8	n.a.	[123]
*P. australasiae*	13	L, n.a.	[22]
	17.1	L, 75	[124]
*P. brunnea*	20.2	L, n.a.	[22]
*P. fuliginosa*	16.7	L, n.a.	[22]
*P. japonica*	9.1–12.2	L, n.a.	[22]

**Table 6:** Threshold temperatures for the individual development (LDT) of selected stored product flies. L—estimated based on linear model, n.a.—data are not available.

Species	LDT (°C)	Model, r.h. (%)	Reference
*Calliphora vicina*	1.0	L, >40	[125]
*C. vomitoria*	6.0	n.a.	[126]
*Chrysomya albiceps*	10.21–15.39	L, n.a.	[127]
*Drosophila melanogaster*	8.2–13.1	L, n.a.	[24]
*D. suzukii*	7.2	L, 60–70	[128]
	8.8	n.a.	[29]
*Fannia canicularis*	0.5	L, n.a.	[24]
*F. femoralis*	4.9	L, n.a.	[24]
*Hydrotaea aenescens*	12.8	n.a.	[29]
*Lucilia cuprina*	8.2	L, n.a.	[24]
	12.02	L, n.a.	[129]
*Megaselia abdita*	6.0	n.a.	[126]
*Musca domestica*	11.0–11.4	L, n.a.	[24]
	12.42	L, 75	[69]
*Muscina stabulans*	4.4–7.8	L, n.a.	[130]
*Ophyra aenescens*	8.9	L, 75–95	[131]
*O. capensis*	13.8	L, 75–95	[131]
*Piophila casei*	9.0	n.a.	[132]
*Phormia regina*	12.2–14.0	L, 50–70	[133]
*Protophormia terraenovae*	9.8	L, 60	[134]
*Psychoda alternata*	7.2	n.a.	[135]
*Sarcophaga argyrostoma*	7.2	L, 60	[136]
*S. bullata*	12	L, n.a.	[24]
*S. tibialis*	5.2	L, n.a.	[137]

**Table 7:** Comparison of lower respiration (LRT) and lower development (LDT) thresholds in stored product mites (LRT—according Hubert et al. [58]; LDT according Table 1).

Species	LDT	LRT
*A. siro*	2.5	2.3
*L. destructor*	3.0	2.9
*T. putrescentiae*	5.0	0.8

**Table 8:** Summary of threshold temperatures for flight (LFT), walking (LWT) and trap capture (LTCT-WP) for stored product insects (Coleoptera, Lepidoptera).

Species	LFT (°C)	Reference	LWT (°C)	Reference	LTCT-WP (°C)	Reference
*Ahasverus advena*	17.5	[44]	7.5	[17]		
*Alphitobius diaperinus*			6.0	[76]		
*Cryptolestes ferrugineus*	22.5	[43]	5.0	[17]	6.3–8.9	[18]
			4.0–8.0	[18]		
*Ephestia elutella*	15.0	[44]				
*E. kuehniella*	12.5	[44]				
*Lasioderma serricorne*	22.5	[145]				
*Oryzaephilus surinamensis*			2.5	[17]		
*Plodia interpunctella*	15.0	[44]				
*Rhyzopertha dominica*	20.0	[44]	7.5	[17]		
	16.0	[144]	7.1	[152]		
*Sitophilus granarius*			2.5	[17]		
*S. oryzae*	27.5	[44]	5.0	[17]	9.5	[18]
	22.0	[153]	9.0	[18]		
*S. zeamais*			9.0	[18]	10.9	[18]
*Tineola bisselliella*	13.0	[102]				
*Tribolium castaneum*	25.0	[44]	10.0	[17]		[18]
			8.0-8.5	[18]	9.3–10.2	[18]
*Trogoderma granarium*			6.5-10.0	[102]		
*T. variabile*	12.0–16.0	[144]				
*Typhaea stercorea*	17.5	[44]				
